# Bovis Bacillus Calmette–Guerin (BCG) infection induces exosomal miRNA release by human macrophages

**DOI:** 10.1186/s12967-017-1205-9

**Published:** 2017-05-12

**Authors:** Shamila D. Alipoor, Esmaeil Mortaz, Payam Tabarsi, Parissa Farnia, Mehdi Mirsaeidi, Johan Garssen, Masoud Movassaghi, Ian M. Adcock

**Affiliations:** 1 Clinical Tuberculosis and Epidemiology Research Center, National Research Institute of Tuberculosis and Lung Diseases (NRITLD), Shahid Beheshti University of Medical Sciences, Tehran, Iran; 2 Department of Biotechnology, School of Advanced Technologies in Medicine, Shahid Beheshti University of Medical Sciences, Tehran, Iran; 3 Institute of Medical Biotechnology, Molecular Medicine Department, National Institute of Genetic Engineering and Biotechnology (NIGEB), Tehran, Iran; 4 Division of Pharmacology, Utrecht Institute for Pharmaceutical Sciences, Faculty of Science, Utrecht University, Utrecht, Netherlands; 5 Department of Immunology, Faculty of Medicine, Shahid Beheshti University of Medical Sciences, Tehran, Iran; 6 Mycobacteriology Research Centre, National Research Institute of Tuberculosis and Lung Disease (NRITLD), Shahid Beheshti University of Medical Sciences, Tehran, Iran; 7 Division of Pulmonary, Critical Care, Sleep and Allergy, Department of Medicine, University of Miami, Coral Gables, FL USA; 8 Nutricia Research Centre for Specialized Nutrition, Utrecht, Netherlands; 9 Department of Pathology and Laboratory Medicine, University of California, Los Angeles (UCLA), Los Angeles, CA USA; 10 Airways Disease Section, National Heart & Lung Institute, Imperial College London, London, UK; 11 Priority Research Centre for Healthy Lungs, Hunter Medical Research Institute, The University of Newcastle, Newcastle, NSW Australia

**Keywords:** Mycobacterium, Exosome, miRNA, Macrophage, Biomarker

## Abstract

**Background:**

Tuberculosis (TB) remains a significant global health concern and its diagnosis is challenging due to the limitations in the specificity and sensitivity of the current diagnostic tests. Exosomes are bioactive 30–100 nm vesicles produced by most cell types and are found in almost all human body fluids. Exosomal microRNAs (miRNAs) can transfer biological information between cells and tissues and may act as potential biomarkers in many diseases. In this pilot study, we assessed the miRNA profile of exosomes released from human monocyte-derived macrophages upon infection with *Mycobacterium bovis* Bacillus Calmette–Guerin (BCG).

**Methods:**

Human monocytes were obtained from the peripheral blood of three healthy subjects and driven to a monocyte-derived macrophage (MDM) phenotype using standard protocols. MDMs were infected with BCG or left uninfected as control. 72 h post-infection, exosomes were collected from the cell culture medium, RNA was isolated and RNA-seq performed. The raw reads were filtered to eliminate adaptor and primer sequences and the sequences were run against the mature human miRNA sequences available in miRBase. MicroRNAs were identified using an E value <0.01. miRNA network analysis was performed using the DIANA miRNA tool, miRDB and functional KEGG pathway analysis.

**Results:**

Infection of MDMs with BCG leads to the release of several exosomal miRNAs. These included miR-1224, -1293, -425, -4467, -4732, -484, -5094, -6848-6849, -4488 and -96 all of which were predicted to target metabolism and energy production-related pathways.

**Conclusions:**

This study provides evidence for the release of specific exosomal miRNAs from BCG-infected MDMs. These exosomal miRNAs reflect host-pathogen interaction and subversion of host metabolic processes following infection.

## Background

Tuberculosis (TB) is a major global threat to human health and despite the availability of effective treatments, it remains a significant health concern especially in low-income countries [1]. The World Health Organization (WHO) reported nearly 10 million new cases and 1.7 million deaths from TB in 2010 [2]. In addition, two billion people worldwide are estimated to live with latent *M. tuberculosis* (M.tb) infection and so act as a potential source of infection [3].

Early TB diagnosis is the key step in controlling the disease in the community and an essential component for preventing the spread of infection. However, this is challenging because of the limitations of the current TB diagnostic methods with respect to their specificity and sensitivity [4–6]. Due to this lack of optimal methods for the detection of TB, the definition of novel biomarkers would be of great practical value. BCG vaccines are live vaccines derived from a strain of *Mycobacterium bovis*, and has been shown that extracellular forms of BCG in the mucosal lymphatic tissues following oral vaccination [7].

Exosomal biomarkers are considered as possible novel diagnostic biomarkers especially in infectious diseases [8–10]. Exosomes are bioactive vesicles of 30–100 nm in diameter, which are secreted from most cell types and can be found in nearly all human bodily fluids [11, 12]. Exosomes enable cell-to-cell communication by shuttling various molecules including miRNAs between cells [12–14]. Different signatures of exosomal content have been reported in various diseases [12, 15–18] including cancers [19–23], acute myeloid leukemia (AML) [24, 25] and Alzheimer’s disease [26].

MicroRNAs are important regulatory molecules that play critical roles in pathological conditions [27, 28]. The role of miRNAs in modulation of innate and adaptive immunity and cellular responses to bacterial infection has been reported previously [29–32]. Various bacterial components, such as peptidoglycan (PG), lipoproteins and lipopolysaccharide (LPS) can affect the host’s miRNA expression levels [33, 34] and trigger inflammatory responses [35]. Functional miRNAs are capsulated in exosomes and delivered to recipient cells and subsequently cause specific modulation of their transcriptome [36]. Several studies have described the role of exosomes in TB [1, 8, 37–39]. Exosomes released from macrophages infected with M.tb, as well as exosomes isolated from M.tb-infected mice, promote both innate and acquired immune responses in vitro and in vivo [1, 8, 37–39]. The modulatory effects of exosomes released from M.tb-infected macrophages has been reviewed [40] and indicate that they can stimulate production of inflammatory mediators and induction of apoptosis in recipient cells [28, 40].

Exosomal miRNAs have been proposed as potential biomarkers in numerous diseases such as cardiovascular disease, malignancies, and Alzheimer’s disease [15, 16, 41–43] although the role of exosomal miRNAs as potential biomarkers TB are not well described. In this pilot study, we profiled the exosomal miRNA of human macrophages after co-infection with *Mycobacterium bovis,* Bacillus Calmette–Guerin (BCG). We hypothesized that BCG-infected macrophages would secrete a specific set of exosomal miRNAs that may playing role in the pathogenesis of TB.

## Methods

### Cell culture

Peripheral blood was obtained from three healthy human donors who had no clinical manifestations of disease. Complete blood count (CBC), ESR, CRP and liver and kidney function tests were evaluated. To investigate prior exposure of TB, QuantiFERON-TB Gold (QFT^®^) and PPD tests were performed and all three healthy subjects were negative for latent TB. An institutional review board (IRB) from Dr. Masih Daneshvari Hospital, Tehran, Iran approved the study.

PBMCs were isolated by density gradient centrifugation using Ficoll-Paque (Invitrogen Corp., Carlsbad, CA). The monocyte-enriched layer was collected from the Ficoll:plasma interface and cells were cultured in Roswell Park Memorial Institute medium (RPMI 1640) (Gibco; Carlsbad, CA, USA) supplemented with 10% heat-inactivated fetal bovine serum (FBS) (Gibco), 25 mM HEPES (Gibco), 100 units/ml penicillin (Sigma, Munich, Germany) and 100 μg/ml streptomycin (Sigma) in 5% CO_2_ at 37 °C. After 4 h, non-adherent cells, which were mostly T lymphocytes, were removed. The adherent monocytes were washed with 1× PBS (Sigma) 2–3 times and were collected by centrifugation for 10 min at 400*g*. Macrophages were obtained by culturing the monocytes for 7–8 days in RPMI containing 100 ng/ml GM-CSF (Invitrogen) and 10% FBS with one medium change with GM-CSF on the 4th day as described previously [44].

### Infection assay

On day 7 or 8, the GM-CSF medium was removed and replaced with fresh medium without GM-CSF for at least 4 h before infection. Uptake of bacteria was assessed by flow cytometry to determine the infection ratio required to obtain 85% infectivity as described previously [45, 46]. Ten flasks each containing 1 × 10^7^ cells were infected with opsonized BCG (obtained as a gift from Pasteur Institute of Iran, IPI) at an MOI (Multiplicity of infection) of 10 or were left uninfected as controls. Cells were incubated for 2 h at 37 °C in a 5% CO_2_ before washing with 1× PBS containing amikacin (80 μg/ml) to eliminate possible free organisms. Subsequently, the cells were incubated in medium containing exosome-depleted FBS (10% final concentration) (System Bioscience, CA, USA) and 100 ng/ml GM-CSF for 72 h.

### Exosome isolation and characterization

Exosomes were isolated from the culture supernatants of infected and uninfected cells 72 h post infection using total exosome isolation (TEI) reagent according to the manufacturer’s instructions (Invitrogen by the Thermo Fisher Scientific corporation, Waltham, MA, USA). Briefly, the cell culture media (CCM) were centrifuged at 300×*g* for 30 min and filtered twice through a 0.22 μm filter (Merck-Millipore, Billerica, MA, USA) to remove apoptotic bodies, dead cells and cell debris. The CCM was mixed with TEI solution at a 5:1 ratio. The samples were incubated overnight at 4 °C and centrifuged for 1 h at 10,000×*g*. The pellet was re-suspended in 1 ml of PBS and stored at −20 °C. Purified exosomes were characterized by electron microscopy (Carl Zeiss NTS, Oberkochen, Germany) and nanoparticle tracking analysis (Malvern/Nanosight LM10. CA, USA).

### Exosomal RNA isolation and qualification

Prior to RNA extraction, exosomes were again filtered through a 0.22 mm filter and treated with RNase A (5 μg/μl Fermentase, Thermo-Fisher, Boston, MA, USA) for 90 min at 37 °C to eliminate non-exosomal RNAs. Total RNA was isolated from exosomes using the total exosomal RNA and protein isolation kit (Thermo-Fisher) according to the manufacturer’s instructions. RNA concentration and purity was measured using a Nano-drop 2000 spectrophotometer (NanoDrop Technologies Inc., Wilmington, DE, USA). The quality, yield, and size of extracted RNA was analyzed using capillary electrophoresis (Agilent 2100 Bioanalyzer, Agilent Technologies, Foster City, CA, USA).

### Small RNA library construction and miRNA sequencing

Size selection and gel purification of the RNA samples was performed on a 15% Tris–Borate-EDTA (TBE) polyacrylamide/urea gel, RNAs were excised from the gel, purified as above and small RNA libraries constructed as described previously [47]. RNA libraries were run on a Roche 454 Genome Sequencer FLX according to the manufacturer’s instructions. The raw reads were filtered to eliminate adaptor sequences and the sequences, in FASTQ format, were run against the mature human miRNA sequence database available at miRBase using BLAST software. MicroRNAs were identified using an E value cutoff <0.01. MicroRNA pathway analysis was performed using the DIANA miRNA tool [48, 49], miRDB [50] and functional KEGG pathway database [51].

## Results

### Exosome characterization and exosomal RNA preparation

**Fig. 1:**
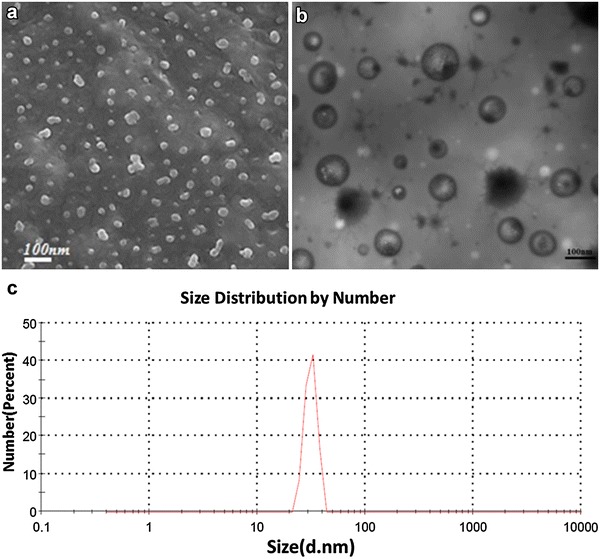
Exosome characterization by scanning (SEM) and transmission electron microscopy (TEM) and nanoanalyzer. Exosomes were isolated by total exosome isolation reagents and passed twice through a 0.22 mm filter before being analyzed for morphology by SEM (**a**) and TEM (**b**). The size distribution of the isolated exosomes was further analyzed by nanoanalyzer (**c**) and showed the size distribution of the exosomes as 70 ± 3.4 nm. The results are representative of three independent experiments

Exosomes were isolated from the culture supernatants from paired infected and uninfected monocyte-derived macrophages from healthy subjects. Exosomes were confirmed by scanning (SEM) and transmission electron microscopy (TEM) and characterized for size distribution by nanoparticle analyzer and demonstrated the expected size and morphology (Fig. 1).

**Fig. 2:**
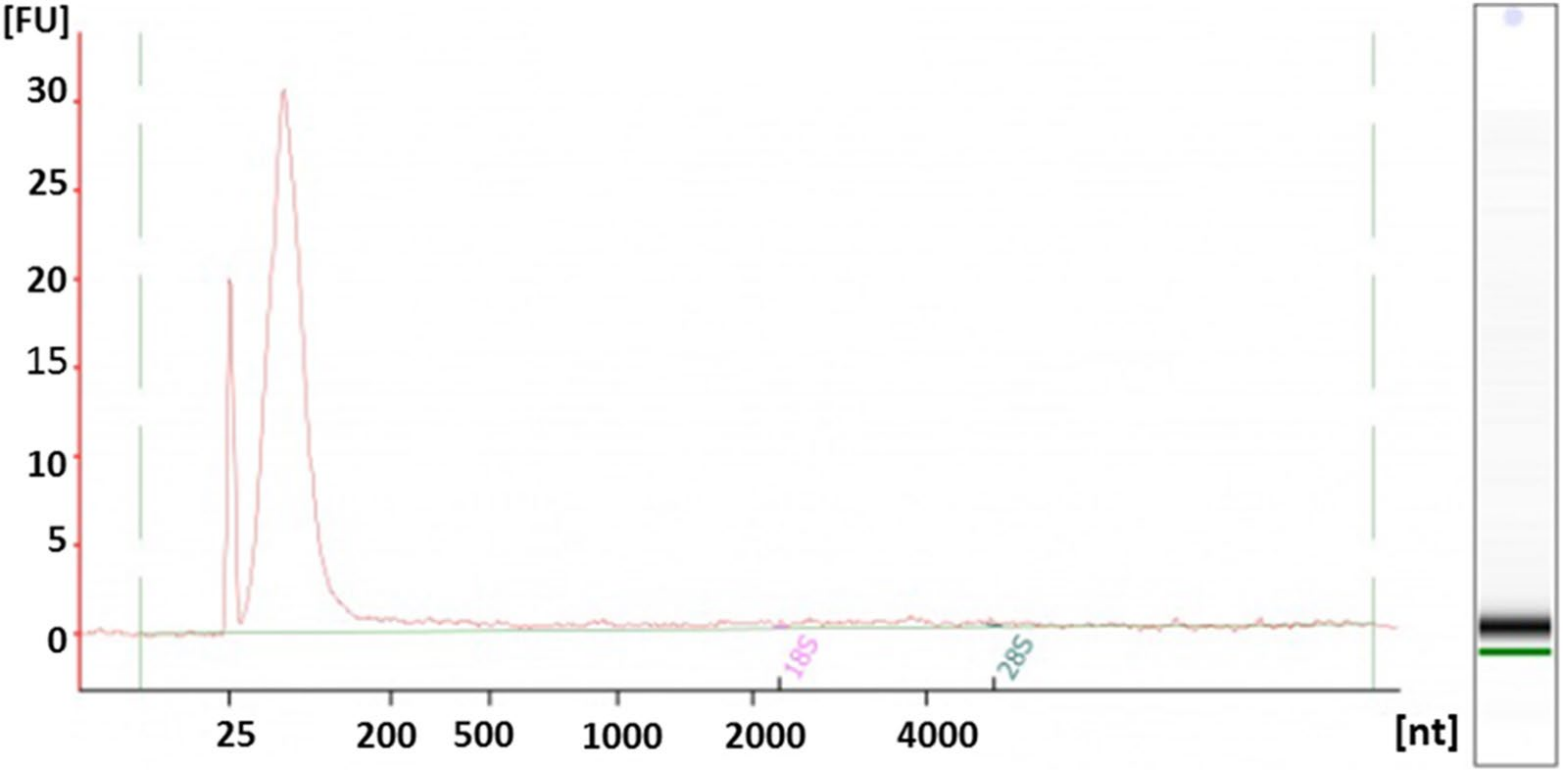
Exosomal RNA qualification. Total RNA was extracted from the exosomes and the quality of the exosomal RNA was assessed by running the samples on an Agilent Bioanalyzer. The RNA population observed in the exosomes were predominantly from small RNAs. The result is representative of three independent experiments

Total exosomal RNA was extracted and the concentration measured. The amount of RNA isolated from 10 T75 culture flasks in each group varied from 1.3 to 1.8 µg from the infected and uninfected cell-derived exosomes with no significant differences between groups (p > 0.05). The extracted exosomal RNA was also qualified on an Agilent Bioanalyzer and the RNA population observed in the exosomes were predominantly from small RNAs (Fig. 2).

### Infection with BCG significantly modulates the miRNA content in infected cell-derived exosomes

Using an E value <0.01 as the cut-off, 44 and 47 miRNAs were identified in the infected and uninfected macrophage-derived exosomes, respectively (Table 1). 38 of these miRNAs (~78%) were present in both infected and uninfected exosomes but 11 miRNAs (miR-1224, -1293, -425, -4467, -4732, -484, -5094, -6848 and -6849, -96 and -4488) were predominantly expressed in exosomes derived from M.tb infected cells. The fold-differences in expression of the 11 miRNAs differentially expressed between infected and non-infected macrophages are shown in Table 2.

**Table 1:** Exosomal miRNA content in infected and uninfected macrophage-derived exosomes

	Infected exosomal miRNAs	Uninfected exosomal miRNA
Common miRNAs according to expression level	mir-486, mir-21, mir-146a, mir-92a, mir-146b, let-7i, mir-423, mir-378a, let-7f, let-7g, let-7a, mir-155, mir-320a, mir-191, let-7b, mir-24-, mir-26a, mir-423, mir-140, mir-30d, mir-148a, mir-101, mir-221, mir-103a, let-7e, mir-28, mir-1307, mir-151a, mir-148b, mir-26b, mir-27a, mir-532, let-7d, mir-361, mir-99b, mir-342, mir-941, mir-511	mir-21, mir-146b, mir-146a, let-7f, let-7i, let-7a, mir-378a, let-7g, let-7b, mir-26a, mir-191, mir-423, mir-30d, mir-155, mir-320a, mir-511, mir-423, mir-1307, mir-101, mir-24, mir-92a, mir-148a, mir-99b, mir-532, let-7d, mir-486, let-7e, mir-103a, mir-221, mir-26b, mir-140, mir-148b, mir-28, mir-151a, mir-941, mir-342, mir-27a, mir-361

Differentially expressed exosomal miRNAs (p < 0.05) obtained from three independent experiments. 44 and 47 miRNAs were identified in the M.tb-infected and -uninfected macrophage-derived exosomes with a copy number >20

**Table 2:** Differentially expressed microRNAs in the exosomes released specifically from M.tb-infected cells

miRNA name	Log Fc	Log CPM	p value	FDR
hsa-miR-96	5.89073	2.930204	0.000245	0.000186
hsa-miR-1224	5.217967	3.015217	0.000519	0.000361
hsa-miR-1293	3.171918	14.19242	0.00203	0.001093
hsa-miR-4467	3.174797	12.62878	0.00204	0.001093
hsa-miR-6848	4.143784	3.760948	0.00578	0.002233
hsa-miR-6849	3.118348	7.30397	0.00826	0.003
hsa-miR-4488	3.184877	5.073045	0.000404	0.010436
hsa-miR-425	3.481839	4.249725	0.000572	0.012611
hsa-miR-4732	2.578799	8.273334	0.000616	0.013178
hsa-miR-484	2.389806	7.714758	0.001566	0.028757
hsa-miR-5094	5.125813	1.680093	0.002864	0.04123

*FC* fold change, *CPM* read per million, *FDR* false discovery rate

### Differentially expressed miRNAs were associated with pathways related to pathogenesis of mycobacterial infection and intracellular survival

To examine the target pathways affected by the differentially expressed miRNAs in the infected macrophage-like group, miRNA network analysis was performed. Pathway analysis showed differential activation of pathways related to mycobacterium invasion, intracellular survival, energy production machinery and immunity reactions (Table 3). Most of the target genes regulated by these miRNAs were involved in the cell infection process and energy production pathways (Table 4). A subgroup of these differentially expressed miRNAs (miR-484, -5094, -425, -1293, -6848, -6849) had the most profound effect on the pathways activated by BCG infection (Table 5).

**Table 3:** Functional pathways identified in mRNA targets for miRNAs

	p value	Number of miRNAs in each pathway
Pathways in bacterial digestion		
Proteasome	0.0139	3
Ubiquitin-mediated proteolysis	0.0057	7
Pathways in infection process		
Bacterial invasion of epithelial cells	0.0310	7
Endocytosis	0.0337	8
ECM-receptor interaction	0.00153	4
Adherents junction	0.00730	4
Inositol phosphate metabolism	0.00201	4
Pathways in fatty acid metabolism		
Fatty acid biosynthesis	0.00000000684	3
Fatty acid metabolism	0.00000154	5
Fatty acid elongation	0.000738	3
Steroid biosynthesis	0.00631	3
2-Oxocarboxylic acid metabolism	0.0165	3
Pathways in amino acids metabolism		
Phenylalanine, tyrosine and tryptophan biosynthesis	0.000713	3
Valine, leucine and isoleucine degradation	0.0310	6
Lysine degradation	0.0421	6
Pathways for energy production and sugar metabolism		
Central carbon metabolism	0.00435	5
2-Oxocarboxylic acid metabolism	0.0165	3
Glycosaminoglycan biosynthesis—heparan sulfate/heparin	0.0113	3
Proteoglycans	0.0337	8
Cell signaling pathways		
Phosphatidylinositol signaling system	0.0489	5
TGF-beta signaling pathway	0.0489	4

**Table 4:** The number of genes targeted by the miRNAs in the altered pathways

Pathways	Gene	p value
Pathways in fatty acid metabolism		
Fatty acid biosynthesis	2	0.000000000684
Fatty acid metabolism	12	0.00000154
Fatty acid elongation	7	0.000738
Steroid biosynthesis	6	0.00631
2-Oxocarboxylic acid metabolism	6	0.0165
Pathways in amino acids metabolism		
Phenylalanine, tyrosine and tryptophan biosynthesis	4	0.000713
Valine, leucine and isoleucine degradation	12	0.0310
Lysine degradation	16	0.0421
Pathways of energy production and sugar metabolism		
Central carbon metabolism in cancer	19	0.00435
2-Oxocarboxylic acid metabolism	6	0.0165
Glycosaminoglycan biosynthesis—heparan sulfate/heparin	9	0.0113
Proteoglycans	49	0.0337
Pathways in infection		
Bacterial invasion of epithelial cells	23	0.0310
Endocytosis	51	0.0337
ECM-receptor interaction	16	0.00153
Adheres junction	21	0.00730
Inositol phosphate metabolism	15	0.0201
Cell signaling pathways		
Phosphatidylinositol signaling system	21	0.0489
TGF-beta signaling pathway	18	0.0489
Pathways in bacterial digestion		
Proteasome	3	0.0138
Ubiquitin mediated proteolysis	7	0.00566

**Table 5:** The number of target genes regulated by the differentially expressed miRNAs in the dysregulated pathways

Pathways	Differentially expressed miRNA	Number target genes
Bacterial invasion pathway	hsa-miR-484	15
	hsa-miR-6848-	4
	hsa-miR-5094	1
	hsa-miR-425-5p	9
	hsa-miR-1293	2
	hsa-miR-4488-5p	
	hsa-miR-1224-5p	1
	hsa-miR-4732-	1
Fatty acid biosynthesis pathways	hsa-miR-1293	1
	hsa-miR-484	1
	hsa-miR-425-5p	1
Fatty acid metabolism	hsa-miR-5094	2
	hsa-miR-425-5p	4
	hsa-miR-1293	3
	hsa-miR-484	4
	hsa-miR-6849-	1
Trp/phe synthesis	hsa-miR-6848-	1
	hsa-miR-4467	1
	hsa-miR-484	2
Fatty acid elongation	hsa-miR-425-5p	4
	hsa-miR-484	2
	hsa-miR-5094	1
Central carbon metabolism	hsa-miR-1224-5p	5
	hsa-miR-484	10
	Has-mir-96	
	hsa-miR-425-5p	6
	hsa-miR-6848-	1
	hsa-miR-6849-	1
Steroid biosynthesis	hsa-miR-484	4
	hsa-miR-4732-	1
	hsa-miR-425-5p	3
Glucose amino glycan	hsa-miR-484	4
	hsa-miR-6848-	2
	hsa-miR-5094	1
	hsa-miR-1293	1
	hsa-miR-1224-5p	1
	hsa-miR-6849-	1
2′-Oxycarboxyl	hsa-miR-484	4
	Has-miR-96	
	hsa-miR-6848-	2
	hsa-miR-5094	1
	hsa-miR-1293	1
	hsa-miR-1224-5p	1
	hsa-miR-6849-	1
Val/leu/isoleu synthesis	hsa-miR-484	5
	hsa-miR-4732-	2
	hsa-miR-5094	3
	hsa-miR-425-5p	1
	hsa-miR-4467	1
	hsa-miR-1293	1
Proteoglycan	hsa-miR-5094	5
	hsa-miR-484	27
	hsa-miR-425-5p	17
	hsa-miR-1293	2
	hsa-miR-1224-5p	4
	hsa-miR-4732-	2
	hsa-miR-6849-	3
	hsa-miR-6848-	4
Lysine degradation	hsa-miR-484	11
	hsa-miR-5094	3
	hsa-miR-1293	4
	hsa-miR-425-5p	4
	hsa-miR-6848-	1
	hsa-miR-1224-5p	1

## Discussion

In the current study, we assessed the exosomal miRNAs released from human macrophages following infection with BCG. We detected a group of 11 exosomal miRNAs miRs-1224, -1293, -425, -4467, -4732, -484, -5094, -6848, -6849, -96 and -4488) that were differentially expressed in infected cells. These miRNAs are involved in several key pathways including central carbon metabolism, fatty acids and sugar metabolism, amino acid metabolism, bacterial invasion related pathways and cell signaling pathways. This suggests that host pathways implicated in immune surveillance are modulated to enable bacterial survival within infected macrophages.

Recent studies have highlighted the role of exosomes as a vehicle for the transfer of proteins, lipids as well as biologically active miRNAs to distant cells. Exosomes may also act as novel biomarkers in several diseases such as acute myeloid leukemia (AML), ovarian cancer, asthma and sarcoidosis [20, 24, 36]. The miRNA profile and some aspects of exosomal content has recently been examined in TB patients [18, 37, 52, 53]. In these studies, the levels of serum free miRNAs [54], macrophage cell miRNAs [55] and exosomal protein content [39] were evaluated. The expression of 14 miRNAs in M.tb-infected macrophages were significantly altered and depended upon the infective strain (Beijing/W or non-Beijing/W strains) [55] and did not overlap with those reported here from M.tb-infected macrophage-derived exosomes. There were no overlapping miRNAs found in the serum of infected patients [54] and the target pathways were distinct from those seen here. This highlights the potential for detecting strain-specific infection using exosomal miRNAs.

Exosomal transport of miRNAs enables their stability and delivery throughout the body [56]. Exposure of cells to various bacterial components affects the host miRNA profile [34] and the this is also evident in the miRNA profile of released exosomes reported in this study. This will potentially cause significant functional modulation of both near and remote recipient cells [33]. Since the nutrients required for efficient M.tb growth and proliferation inside the host cell are restricted [57], bacteria sequester the host’s cell metabolism by activation of virulence-associated factors [57]. These may trigger regulatory miRNA networks that control the carbon and nitrogen metabolism in cells [58, 59] and also determine their release into exosomes.

Fatty acid biosynthesis and associated downstream metabolites may be affected by exosomal miRNAs induced by BCG treatment. Thus, one of the dysregulated exosomal miRNAs found in this study, miR-1224, is involved in the regulation of lipid metabolism. miR-1224 regulates the expression of lipid-related genes that are directly regulated by transcription factors such as SP1 [60]. In support of this, studies have shown an abundance of lipids in caseous pulmonary granulomas from TB patients [61]. Transcriptome analysis of these granulomas demonstrated a significant up-regulation of genes involved in the sequestration, catabolism, and synthesis of host lipids [61]. Moreover, an increased level of TNF-α in response to exposure to M.tb cell wall components, upregulates the activation of genes related to lipid metabolism in infected cells [61]. MicroR-1224 has a negative regulatory effect on TNF-α gene expression [35] suggesting an interaction between these pathways. Overall, it is evident that alteration of the host’s lipid metabolism plays a crucial role the survival of intracellular M.tb [61–63]. M.tb uses the host’s fatty acids as a source of carbon and to limit the effect of propionyl-CoA, a potentially toxic intermediate, has on its own survival. The metabolism of host lipids could lead to the expansion of the acetyl CoA pool in infected cells [64].

The dysregulated microRNAs reported here also target central carbon metabolism (CCM) pathways. CCM transforms carbon to energy via glycolysis, gluconeogenesis, the pentose phosphate pathway and the tricarboxylic acid cycle (TCA) pathways [65]. The pathogenicity of M.tb depends on the reprogramming of the host cells’ metabolic pathways [62, 66–68]. Moreover, CCM plasticity determines pathogen adaptation to the intercellular milieu of the macrophage [58]. The macrophage glycolytic flux is disturbed by M.tb infection which results in increased glucose uptake by the pathogen-infected cell [57]. Metabolomic profiling using H-NMR of mycobacterial-infected lung tissue showed significant changes in the host cell’s metabolic pathways [69, 70]. Mtb infection decreases the intracellular levels of glucose, glycogen, NAD and NADP whilst increasing the levels of lactate. This reduced level of TCA cycle intermediates in infected tissues is associated with enhanced aerobic glycolysis resulting from a pentose-phosphate shunt and enhanced uptake of glucose [70].

Dysregulated miRNAs released into exosomes from BCG-infected macrophages also affected amino acid synthesis and metabolism pathways (Table 3). Metabolic profiling demonstrated increased levels of amino acids and activation of pyrimidine and purine nucleotide biosynthesis within M.tb-infected lung tissue [71].

Another group of differentially expressed miRNAs in exosomes from infected macrophages were associated with cell membrane and communication pathways such as adherens junction, gap junction, glycosaminoglycan biosynthesis and heparan sulfate/keratin sulfate metabolism. MicroR-1293 for example targets the tissue inhibitors of metalloproteinases (TIMPs) [72]. TIMP-1 is an inhibitor of matrix metalloproteinases and is involved in the invasion and spreading of bacteria through the epithelial cell [73]. M.tb infection up-regulates the expression of matrix metalloproteinases (MMP) and perturbs the MMP/TIMP balance in human monocytes [74]. The up-regulation of miR-1293 in exosomes from BCG-infected macrophages may reflect the ability of mycobacterium antigens to alter the host cell membrane structure and subsequently affect macrophage survival.

miR-484 and miR-425 were preferentially found in exosomes of BCG-infected macrophages. miR-484 regulates intermediate metabolic pathways by targeting the mitochondrial fission protein 1 (Fis1) [75] and altered miR-425 expression is linked to insulin resistance. The role of these miRNAs in infected macrophages remains unclear at this point although it is evident that miR-425 regulates several metabolic pathways and has been associated with metabolic disorders [76].

miRs-1224, -1293, -4467, -4732, -5094, -6848 and -6849 are human mirtrons which are produced via splicing of introns from mRNA coding genes rather than by the formation of hairpin loops by Drosha [77]. The expression of these mirtrons were significantly higher in the exosomes released from infected macrophages. This suggests that mycobacteria may recognize, at least in part, the pattern of miRNA production within the host cell and program over-expression of these mirtrons in order to recruit host metabolic pathways that favour M.tb infection.

## Conclusion

This pilot study demonstrated the differential expression of many miRNAs within exosomes released from BCG-infected macrophages. These miRNAs indicate that metabolic reprogramming may occur to favour M.tb survival. Further studies are needed in large cohorts of patients to test for the presence of these 11 miRNAs in blood exosomes to determine their true value as a possible diagnostic biomarker for TB infection. The profiling of miRNAs upon BCG infection may shed additional light on the host-pathogen interaction and changes in cellular function. Future studies on miRNA expression and function in TB may provide greater understanding of M.tb pathogenesis.

## Abbreviations

BCG: Bovis Bacillus Calmette–Guerin
CCM: central carbon metabolism
FBS: fetal bovine serum
LPS: lipopolysaccharide
MDM: monocyte-derived macrophages
MMP: matrix metalloproteinases
miRNA: microRNAs
QuantiFERON-TB Gold (QFT^®^): interferon-gamma (IFN-y) release assay
PPD: purified protein derivative
TEM: transmission electron microscopy
TB: tuberculosis

## References

[1] Kruh-Garcia NA, Wolfe LM, Dobos KM (2015). Deciphering the role of exosomes in tuberculosis. Tuberculosis.

[2] Organization, W.H. (2015). Global tuberculosis report 2015.

[3] Person AK, Pettit AC, Sterling TR (2013). Diagnosis and treatment of latent tuberculosis infection: an update. Curr Respir Care Rep.

[4] Tsara V, Serasli E, Christaki P (2009). Problems in diagnosis and treatment of tuberculosis infection. Hippokratia.

[5] Davies P, Pai M (2008). The diagnosis and misdiagnosis of tuberculosis. [State of the art series. Tuberculosis. Edited by ID Rusen. Number 1 in the series]. Int J Tuberc Lung Dis.

[6] Babady N, Wengenack N. Clinical laboratory diagnostics for *Mycobacterium tuberculosis*, understanding tuberculosis-global experiences and innovative approaches to the diagnosis. In: Cardona PJ, editor. Rijeka: InTech; 2012. Accessed 10 Oct 2013.

[7] Czepluch W, Dunn AC, Everitt CL, Dorer D, Saunderson SC, Aldwell FE, McLellan AD (2013). Extracellular forms of *Mycobacterium bovis* BCG in the mucosal lymphatic tissues following oral vaccination. Int J Mycobacteriol.

[8] Schorey JS, Harding CV (2016). Extracellular vesicles and infectious diseases: new complexity to an old story. J Clin Investig.

[9] Kruh-Garcia NA, Schorey JS, Dobos KM (2012). Exosomes: new tuberculosis biomarkers-prospects from the bench to the clinic.

[10] Schorey JS, Dobos KM. Exosomes and diagnostic biomarkers. 2016. Google Patents.

[11] Raposo G, Stoorvogel W (2013). Extracellular vesicles: exosomes, microvesicles, and friends. J Cell Biol.

[12] Frydrychowicz M (2015). Exosomes-structure, biogenesis and biological role in non-small-cell lung cancer. Scand J Immunol.

[13] Lin J, et al. Exosomes: novel biomarkers for clinical diagnosis. Sci World J. 2015;2015:657086. doi:10.1155/2015/657086. 10.1155/2015/657086 PMC4322857 25695100

[14] Kourembanas S (2015). Exosomes: vehicles of intercellular signaling, biomarkers, and vectors of cell therapy. Annu Rev Physiol.

[15] Khalyfa A, Gozal D (2014). Exosomal miRNAs as potential biomarkers of cardiovascular risk in children. J Transl Med.

[16] Hu G, Drescher KM, Chen X (2012). Exosomal miRNAs: biological properties and therapeutic potential. Frontiers in genetics.

[17] Jakobsen KR, et al. Exosomal proteins as potential diagnostic markers in advanced non-small cell lung carcinoma. J Extracell Vesicles. 2015;4:26659. doi:10.3402/jev.v4.26659. 10.3402/jev.v4.26659 PMC4348413 25735706

[18] Zhang J (2015). Exosome and exosomal microRNA: trafficking, sorting, and function. Genom Proteom Bioinform.

[19] Zöller M. Exosomes in cancer disease. Cancer gene profiling: methods and protocols. 2016. p. 111–49. 10.1007/978-1-4939-3204-7_7 26667458

[20] Taylor DD, Gercel-Taylor C (2008). MicroRNA signatures of tumor-derived exosomes as diagnostic biomarkers of ovarian cancer. Gynecol Oncol.

[21] Cazzoli R (2013). MicroRNAs derived from circulating exosomes as non-invasive biomarkers for screening and diagnose lung cancer. J Thorac Oncol.

[22] Sun T (2015). Role of exosomal noncoding RNAs in lung carcinogenesis. Biomed Res Int.

[23] Nilsson J (2009). Prostate cancer-derived urine exosomes: a novel approach to biomarkers for prostate cancer. Br J Cancer.

[24] Hornick NI (2015). Serum exosome MicroRNA as a minimally-invasive early biomarker of AML. Sci Rep.

[25] Hornick NI. Exosome trafficking in the acute myeloid leukemia microenvironment. 2015.

[26] Van Giau V, An SSA (2016). Emergence of exosomal miRNAs as a diagnostic biomarker for Alzheimer’s disease. J Neurol Sci.

[27] Zhang X, Dong H, Tian Y. miRNA biology in pathological processes. In: MicroRNA detection and pathological functions. Berlin: Springer; 2015. p. 7–22.

[28] Alipoor SD (2016). The roles of miRNAs as potential biomarkers in lung diseases. Eur J Pharmacol.

[29] Bhatnagar S (2007). Exosomes released from macrophages infected with intracellular pathogens stimulate a proinflammatory response in vitro and in vivo. Blood.

[30] Eulalio A, Schulte L, Vogel J (2012). The mammalian microRNA response to bacterial infections. RNA Biol.

[31] Maudet C, Mano M, Eulalio A (2014). MicroRNAs in the interaction between host and bacterial pathogens. FEBS Lett.

[32] Staedel C, Darfeuille F (2013). MicroRNAs and bacterial infection. Cell Microbiol.

[33] Baltimore D (2008). MicroRNAs: new regulators of immune cell development and function. Nat Immunol.

[34] Moschos SA (2007). Expression profiling in vivo demonstrates rapid changes in lung microRNA levels following lipopolysaccharide-induced inflammation but not in the anti-inflammatory action of glucocorticoids. BMC Genom.

[35] Niu Y (2011). Lipopolysaccharide-induced miRNA1224 negatively regulates tumour necrosis factor-α gene expression by modulating Sp1. Immunology.

[36] Alipoor SD, et al. Exosomes and exosomal miRNA in respiratory diseases. Mediat Inflamm. 2016;2016:5628404. doi:10.1155/2016/5628404. 10.1155/2016/5628404 PMC5055958 27738390

[37] Kruh-Garcia NA (2014). Detection of *Mycobacterium tuberculosis* peptides in the exosomes of patients with active and latent *M. tuberculosis* infection using MRM-MS. PLoS ONE.

[38] Singh PP (2011). Exosomes released from *M. tuberculosis* infected cells can suppress IFN-γ mediated activation of naive macrophages. PLoS ONE.

[39] Giri PK (2010). Proteomic analysis identifies highly antigenic proteins in exosomes from *M. tuberculosis*-infected and culture filtrate protein-treated macrophages. Proteomics.

[40] Singh PP, Li L, Schorey JS (2015). Exosomal RNA from *Mycobacterium tuberculosis*—infected cells is functional in recipient macrophages. Traffic.

[41] Rabinowits G (2009). Exosomal microRNA: a diagnostic marker for lung cancer. Clin Lung Cancer.

[42] Eldh M. Exosomes and exosomal RNA—a way of cell-to-cell communication. 2013.

[43] Sun T, et al. Role of exosomal noncoding RNAs in lung carcinogenesis. BioMed Res Int. 2015;2015:125807. doi:10.1155/2015/125807. 10.1155/2015/125807 PMC4637011 26583084

[44] Sarir H (2009). Cigarette smoke regulates the expression of TLR4 and IL-8 production by human macrophages. J Inflamm.

[45] Busetto S (2004). A single-step, sensitive flow cytofluorometric assay for the simultaneous assessment of membrane-bound and ingested *Candida albicans* in phagocytosing neutrophils. Cytom Part A.

[46] Lehmann AK, Sørnes S, Halstensen A (2000). Phagocytosis: measurement by flow cytometry. J Immunol Methods.

[47] Malone C (2012). Preparation of small RNA libraries for high-throughput sequencing. Cold Spring Harb Protoc.

[48] Reczko M (2012). Functional microRNA targets in protein coding sequences. Bioinformatics.

[49] Paraskevopoulou MD (2013). DIANA-microT web server v5.0: service integration into miRNA functional analysis workflows. Nucleic Acids Res.

[50] Wang X (2008). miRDB: a microRNA target prediction and functional annotation database with a wiki interface. Rna.

[51] Kanehisa M, Goto S (2000). KEGG: kyoto encyclopedia of genes and genomes. Nucleic Acids Res.

[52] Bhatnagar S, Schorey JS (2007). Exosomes released from infected macrophages contain *Mycobacterium avium* glycopeptidolipids and are proinflammatory. J Biol Chem.

[53] Wang JJ (2014). Proteomic analysis and immune properties of exosomes released by macrophages infected with *Mycobacterium avium*. Microbes Infect.

[54] Wang C (2011). Comparative miRNA expression profiles in individuals with latent and active tuberculosis. PLoS ONE.

[55] Zheng L (2015). Differential microRNA expression in human macrophages with *Mycobacterium tuberculosis* infection of Beijing/W and non-Beijing/W strain types. PLoS ONE.

[56] Gallo A (2012). The majority of microRNAs detectable in serum and saliva is concentrated in exosomes. PLoS ONE.

[57] Mehrotra P (2014). Pathogenicity of *Mycobacterium tuberculosis* is expressed by regulating metabolic thresholds of the host macrophage. PLoS Pathog.

[58] Eisenreich W (2013). Metabolic host responses to infection by intracellular bacterial pathogens. Front Cell Infect Microbiol.

[59] Smith I (2003). *Mycobacterium tuberculosis* pathogenesis and molecular determinants of virulence. Clin Microbiol Rev.

[60] Vickers KC (2013). Complexity of microRNA function and the role of isomiRs in lipid homeostasis. J Lipid Res.

[61] Kim MJ (2010). Caseation of human tuberculosis granulomas correlates with elevated host lipid metabolism. EMBO Mol Med.

[62] Daniel J (2011). *Mycobacterium tuberculosis* uses host triacylglycerol to accumulate lipid droplets and acquires a dormancy-like phenotype in lipid-loaded macrophages. PLoS Pathog.

[63] Peyron P (2008). Foamy macrophages from tuberculous patients’ granulomas constitute a nutrient-rich reservoir for *M. tuberculosis* persistence. PLoS Pathog.

[64] Lee W (2013). Intracellular *Mycobacterium tuberculosis* exploits host-derived fatty acids to limit metabolic stress. J Biol Chem.

[65] Rhee KY (2011). Central carbon metabolism in *Mycobacterium tuberculosis*: an unexpected frontier. Trends Microbiol.

[66] Munoz-Elias EJ, McKinney JD (2005). *Mycobacterium tuberculosis* isocitrate lyases 1 and 2 are jointly required for in vivo growth and virulence. Nat Med.

[67] Pandey AK, Sassetti CM (2008). Mycobacterial persistence requires the utilization of host cholesterol. Proc Natl Acad Sci USA.

[68] Rohde KH (2012). Linking the transcriptional profiles and the physiological states of *Mycobacterium tuberculosis* during an extended intracellular infection. PLoS Pathog.

[69] Shin J-H (2011). 1H NMR-based metabolomic profiling in mice infected with *Mycobacterium tuberculosis*. J Proteome Res.

[70] Somashekar B (2011). Metabolic profiling of lung granuloma in *Mycobacterium tuberculosis* infected guinea pigs: ex vivo 1H magic angle spinning NMR studies. J Proteome Res.

[71] MacAllan DC (1998). Whole body protein metabolism in human pulmonary tuberculosis and undernutrition: evidence for anabolic block in tuberculosis. Clin Sci.

[72] Li P (2013). Identification of miR-1293 potential target gene: TIMP-1. Mol Cell Biochem.

[73] Friedland J (2002). Differential regulation of MMP-1/9 and TIMP-1 secretion in human monocytic cells in response to *Mycobacterium tuberculosis*. Matrix Biol.

[74] Elkington PT (2005). *Mycobacterium tuberculosis* up-regulates matrix metalloproteinase-1 secretion from human airway epithelial cells via a p38 MAPK switch. J Immunol.

[75] Wang K (2012). miR-484 regulates mitochondrial network through targeting Fis1. Nat Commun.

[76] Barwari T, Skroblin P, Mayr M (2016). When sweet turns salty: glucose-induced suppression of atrial natriuretic peptide by microRNA-425. J Am Coll Cardiol.

[77] Butkyte S (2016). Splicing-dependent expression of microRNAs of mirtron origin in human digestive and excretory system cancer cells. Clin Epigenetics.

